# Prognostic Role of Serum Amino Acids in Head and Neck Cancer

**DOI:** 10.1155/2020/2291759

**Published:** 2020-10-01

**Authors:** Gabriella Cadoni, Luca Giraldi, Carlo Chiarla, Jacopo Gervasoni, Silvia Persichilli, Aniello Primiano, Stefano Settimi, Jacopo Galli, Gaetano Paludetti, Dario Arzani, Stefania Boccia, Ivo Giovannini, Giovanni Almadori

**Affiliations:** ^1^Dipartimento di Scienze dell'Invecchiamento, Neurologiche, Ortopediche e della Testa-Collo, Fondazione Policlinico Universitario A. Gemelli IRCCS, Largo Gemelli 8, I-00168 Rome, Italy; ^2^Istituto di Clinica Otorinolaringoiatrica, Università Cattolica del Sacro Cuore, Rome, Italy; ^3^Sezione di Igiene, Dipartimento Universitario di Scienze della Vita e Sanità Pubblica, Università Cattolica del Sacro Cuore, Rome, Italy; ^4^Dipartimento Scienze di Laboratorio e Infettivologiche, Fondazione Policlinico Universitario A. Gemelli IRCCS, Rome, Italy; ^5^Dipartimento di Scienze Biotecnologiche di Base, Cliniche Intensivologiche e Perioperatorie, Università Cattolica del Sacro Cuore, Rome, Italy; ^6^Dipartimento della Salute della Donna, del Bambino e di Sanità Pubblica, Area di Sanità Pubblica, Fondazione Policlinico Universitario A. Gemelli IRCCS, Rome, Italy

## Abstract

**Introduction:**

Serum amino acid (AA) profiles represent a valuable tool in the metabolic assessment of cancer patients; still, information on the AA pattern in head and neck cancer (HNC) patients is insufficient. The aim of the study was to assess whether serum AA levels were associated with the stage of neoplastic disease and prognosis in primary HNC patients.

**Methods:**

Two hundred and two primary HNC patients were included in the study. Thirty-one AAs and derivatives were measured in serum through an ultraperformance liquid chromatography-mass spectrometry (UPLC-MS). The association between AA concentrations and the stage (advanced versus early) of HNC was estimated using a multivariable logistic regression model. A multivariable Cox regression model was used to evaluate the prognostic significance of each AA.

**Results:**

At the multivariable logistic regression analysis, increased levels of alpha-aminobutyric acid, aminoadipic acid, histidine, proline, and tryptophan were associated with a reduced risk of advanced stage HNC, while high levels of beta-alanine, beta-aminobutyric acid, ethanolamine, glycine, isoleucine, 4-hydroxyproline, and phenylalanine were associated with an increased risk of advanced stage HNC. Furthermore, at multivariate analysis, increased levels of alpha-aminobutyric acid were associated with increased overall survival (OS), while high levels of arginine, ethanolamine, glycine, histidine, isoleucine, 4-hydroxyproline, leucine, lysine, 3-methylhistidine, phenylalanine, and serine were associated with decreased OS.

**Conclusions:**

Our study suggests that AA levels are associated with the stage of disease and prognosis in patients with HNC. More study is necessary to evaluate if serum AA levels may be considered a hallmark of HNC and prove to be clinically useful markers of disease status and prognosis in HNC patients.

## 1. Introduction

Head and neck cancers (HNCs) are highly aggressive, multifactorial tumors affecting more than 800,000 new patients worldwide each year [1]. Tobacco and alcohol are the main risk factors for HNCs, with human papillomavirus type 16 (HPV16) being a well-established risk factor for oropharyngeal cancer [2]. Furthermore, specific nutrient deficiencies (vitamin C, folates, and carotenoids), food groups, and dietary behaviors are related to the risk for HNC [3, 4].

The majority of HNC present with advanced stage (III-IV) at the time of diagnosis [5]. Treatment involves the standard therapy options of surgery, radiotherapy, and chemotherapy, but all modalities are largely ineffective. Following diagnosis of HNC, 5-year survival varies substantially across countries. In Europe, five-year age standardized relative survival is the highest for laryngeal cancer and the poorest for hypopharyngeal cancer (59% for larynx, 45% for oral cavity, 39% for oropharynx, and 25% for hypopharynx). The risk of death is approximately 2-3 times greater among patients with stage III-IV, respectively, than those with stage I at diagnosis. While HNC can be often cured when diagnosed at an early stage, late-stage disease may be untreatable or involves aggressive multimodality treatment that often leads to severe physical and psychological disabilities [6].

Recently, metabolomics has been used to identify changes in metabolite profiles in the different stages of cancer in order to introduce new noninvasive molecular tools for staging [7].

The past decade has seen rapid advances in our understanding of the metabolic reprogramming that occurs during tumorigenesis. It is well established that tumors display different metabolic phenotypes than normal tissues [8]. Cancer cells develop an altered nutrient utilization to obtain metabolic fuels. Glucose and then glutamine are the most rapidly consumed nutrients by many cultured cancer cell lines [9]. In addition to glucose, there has also been a long-standing interest in understanding the unique amino acid (AA) requirements of cancer cells [10]. Indeed, like glucose, there are major differences in the uptake and secretion of several AAs in tumors relative to normal tissues [11]. Furthermore, it is now appreciated that AAs, rather than glucose, account for the majority of the carbon-based biomass production in rapidly proliferating cancer cells [12]. AAs also contain nitrogen and have been demonstrated to be the dominant nitrogen source for hexosamines, nucleotides, and other nitrogenous compounds in rapidly proliferating cells [13]. Because of these important roles in tumor metabolism, there continues to be significant interest in studying AA metabolism for cancer marker detection. Several investigators have reported changes in peripheral blood AAs in various types of cancer patients, including lung, ovarian, and also HNC patients [14, 15]. The only study which specifically addressed the link between the basal serum AA levels and long-term prognosis in HNC patients concluded that increased serum levels of methionine had a favourable prognostic impact in terms of overall survival (OS) and relapse-free survival, while increased serine showed borderline significance as a negative prognostic factor for OS [15].

The aim of this study was to quantitate a wider panel of serum AAs in primary HNC patients using ultraperformance liquid chromatography-mass spectrometry (UPLC-MS) in order to provide an expanded view of the associations between increased AA levels, stage of disease, and OS.

## 2. Methods

Subjects with histologically confirmed primary squamous cell carcinoma of the head and neck were included. The study was approved by the Ethical Committee of the Policlinico Universitario Agostino Gemelli (protocol ID 2435). HNC tumors were classified into anatomic site according to the following ICD-0-2 categories: oral cavity, oropharynx, hypopharynx, larynx, and not specified site. The tumors were staged according to the tumor, node, metastasis (TNM) classification [16]. The recruitment was conducted from 2002 to 2012 in Rome (Institute of Otorhinolaryngology, Università Cattolica Sacro Cuore, Fondazione Policlinico Universitario Agostino Gemelli). A total of 202 patients gave their consent to have blood withdrawn and processed for research studies and also had a 5-year follow-up. The characteristics of these patients (rates of sex, tumor sites, etc.) did not significantly differ from those of the total number of cases treated in the same period.

### 2.1. Blood Samples

Blood samples were obtained by venipuncture and collected in 8 mL serum collection tubes Vacuette (VACUETTE® TUBE Greiner Bio-One, Kremsmünster, Austria). Every sample was taken once in each patient prior to any treatment. The blood samples were centrifuged at 2000 g at 4°C for 10 min within 60 min after collection. Serum was aliquoted and stored at −80°C until analysis.

### 2.2. Data Collection

Patients were interviewed face-to-face by medical doctors, on demographics and alcohol and tobacco consumption. Participants were followed from the date of diagnosis to the date of death or end of follow-up at June 2017, whichever occurred first. Death certificate data were also used for mortality, and the cause of death was coded according the International Classification of Diseases, Ninth Revision. Data on tumor pathology and treatment were obtained from pathology records.

### 2.3. Ultraperformance Liquid Chromatography-Mass Spectrometry (UPLC-MS)

Thirty-one AAs and derivatives (alanine, alpha-aminobutyric acid, aminoadipic acid, anserine, arginine, asparagine, beta-alanine, beta-aminobutyric acid, carnosine, citrulline, cystathionine, ethanolamine, gamma-aminobutyric acid, glycine, histidine, isoleucine, 4-hydroxyproline, leucine, lysine, methionine, 1-methylhistidine, 3-methylhistidine, phenylalanine, phosphoethanolamine, proline, sarcosine, serine, threonine, tryptophan, tyrosine, and valine) were measured in serum through an ultraperformance liquid chromatography-mass spectrometry (UPLC-MS) validated methodology. Briefly, 50 *μ*L of sample was mixed with 100 *μ*L 10% (*w*/*v*) sulfosalicylic acid containing an internal standard mix (50 *μ*M) and centrifuged at 1000 × g for 15 min. 10 *μ*L of the supernatant was transferred into a vial containing 70 *μ*L of borate buffer to which 20 *μ*L of AccQ Tag reagents (Waters Corporation, Milford, MA) was subsequently added. Samples were then vortexed for 10 s and heated at 55°C for 10 min. The chromatographic separation was performed by ACQUITY H-Class (Waters Corporation) using an ACQUITY CORTECS C18 column (Waters Corporation) eluted at a flow rate of 500 *μ*L/min with a linear gradient (9 min) from 99 to 1 water 0.1% formic acid in acetonitrile 0.1% formic acid. MS was an ACQUITY QDa single quadrupole equipped with electrospray source operating in positive mode (Waters Corporation). Analytical process was monitored using Kairos™ Amino Acid Quality Control (level 1 and level 2) manufactured by Waters.

### 2.4. Statistical Analysis

Descriptive statistics were conducted to describe the study participants. Categorical variables were reported as absolute frequencies and percentages, and continuous variables were reported as mean and standard deviation. Tumor stage was categorized as early stage (I and II) and advanced stage (III and IV). Smoking (and drinking) habits were categorized as never, former, and current smoker (or drinker). OS was defined as the time from the date of diagnosis to the date of death. AA concentrations were dichotomized according to cut-off points calculated by maximally selected rank statistics [17]. A multivariable logistic regression model was used to assess the association with the risk of advanced versus early stage HNC. A multivariable Cox regression model was used to evaluate the prognostic significance of each AA, including the following terms: age, sex, tumor stage, treatment type, smoking status, and alcohol-drinking status. The Kaplan-Meier method was used to plot the survival curves. Statistical analyses were conducted using Stata software (Stata Statistical Software Release 16; StataCorp LP, College Station, Texas, USA) and the R packages Survival [18] and Maxstat [19]. All tests were two sided, and a *p* value < 0.05 was considered as statistically significant.

## 3. Results

### 3.1. Participants' Characteristics

Participants' demographic, clinical, and behavioral characteristics are reported in Table 1. Two hundred and two participants were included in the study, with a higher prevalence of males (75.2%) and an average age of 63 years old. A higher prevalence of participants had a laryngeal tumor subsite (52.0%) and an advanced stage of tumor (64.4%). A higher prevalence of participants underwent surgery and radio- or radio/chemotherapy treatment (38.6%), followed by surgery only (30.7%) and by radio/chemotherapy or radiotherapy (30.2%). A higher prevalence of participants was categorized as former smokers (61.4%) and as current drinkers (67.3%).

### 3.2. Association between AA Serum Levels and Risk for Advanced Stage HNC

In Table 2, we reported the odds ratios (ORs) and 95% CI for the associations between the AA concentrations and the risk of advanced stage of HNC. High levels of alpha-aminobutyric acid (OR: 0.40; 95% CI: 0.16-0.98), aminoadipic acid (OR: 0.36; 95% CI: 0.17-0.78), histidine (OR: 0.40; 95% CI: 0.19-0.85), proline (OR: 0.24; 95% CI: 0.09-0.62), and tryptophan (OR: 0.41; 95% CI: 0.22-0.76) were associated with a reduced risk of advanced stage HNC, while high levels of beta-alanine (OR: 2.55; 95% CI: 1.17-5.60), beta-aminobutyric acid (OR: 1.91; 95% CI: 1.00-3.65), ethanolamine (OR: 2.25; 95% CI: 1.20-4.24), glycine (OR: 2.35; 95% CI: 1.17-4.73), isoleucine (OR: 2.39; 95% CI: 1.20-4.76), 4-hydroxyproline (OR: 3.16; 95% CI: 1.02-9.78), and phenylalanine (OR: 2.72; 95% CI: 1.42-5.21) were associated with an increased risk of advanced stage HNC.

### 3.3. Association between AA Serum Levels and OS

**Figure 1:**
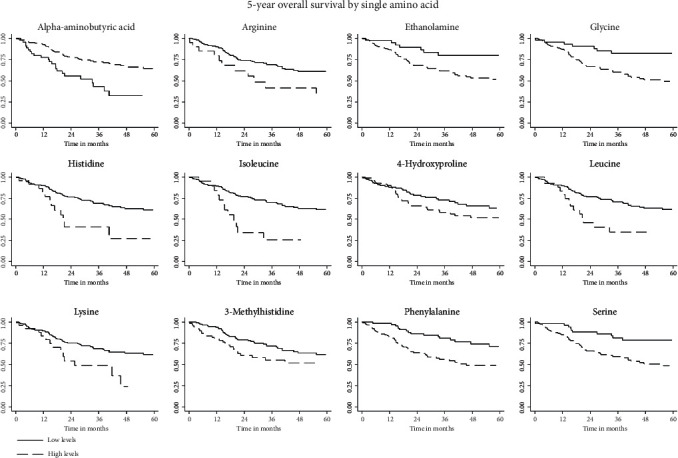
Kaplan-Meier curves for amino acids whose levels were significantly associated with OS.

In Table 3, we reported the HRs and 95% CI for the association between the AA concentrations and the OS. High levels of alpha-aminobutyric acid (HR: 0.41; 95% CI: 0.24-0.69) were associated with increased OS, while high levels of arginine (HR: 2.13; 95% CI: 1.07-4.24), ethanolamine (HR: 2.28; 95% CI: 1.07-4.84), glycine (HR: 2.49; 95% CI: 1.17-5.32), histidine (HR: 2.98; 95% CI: 1.44-6.19), isoleucine (HR: 3.08; 95% CI: 1.49-6.40), 4-hydroxyproline (HR: 1.66; 95% CI: 1.00-2.74), leucine (HR: 2.29; 95% CI: 1.17-4.48), lysine (HR: 2.34; 95% CI: 1.19-4.60), 3-methylhistidine (HR: 1.83; 95% CI: 1.07-3.13), phenylalanine (HR: 2.06; 95% CI: 1.19-3.57), and serine (HR: 2.71; 95% CI: 1.39-5.31) (Figure 1) were associated with decreased OS.

### 3.4. Comparison of AA Levels among Tumor Subsites

In Table 4, we reported the *p* value obtained from the comparison of the AA levels among HNC tumor subsites. A significant difference was observed for ethanolamine levels (*p* = 0.026) and phenylalanine levels (*p* = 0.003). Post hoc comparison showed higher levels of ethanolamine in patients with oral cavity cancer compared to those with larynx (*p* = 0.013) cancer and higher levels of phenylalanine in patients with oral cavity cancer compared to those with hypopharynx (*p* = 0.029) and larynx (*p* = 0.002) cancers (data not shown).

## 4. Discussion

Various factors can influence serum AA pattern in cancer, depending on the type and the stage of the disease: muscle wasting, malnutrition, systemic inflammation, and insulin resistance [20, 21]. To our knowledge, only one study has specifically addressed the link between the basal serum AA levels and long-term prognosis in HNC patients [15]. Our study was done on a larger number of patients, with a less unbalanced female/male patient ratio, measuring a wider panel of serum AAs and evaluating the impact of AA profiles on both the OS and the risk of advanced stage. In our group of patients with HNC, we found that higher serum levels of alpha-aminobutyric acid, aminoadipic acid, histidine, proline, and tryptophan were associated with a reduced risk of advanced stage HNC, while high levels of beta-alanine, beta-aminobutyric acid, ethanolamine, glycine, isoleucine, 4-hydroxyproline, and phenylalanine were associated with an increased risk of advanced stage HNC. Moreover, at multivariate analysis, increased levels of alpha-aminobutyric acid were associated with increased OS, while high levels of arginine, ethanolamine, glycine, histidine, isoleucine, 4-hydroxyproline, leucine, lysine, 3-methylhistidine, phenylalanine, and serine were associated with decreased OS.

It has been suggested that increased muscle breakdown in cancer provides substrates for enhanced gluconeogenesis in the liver and enhanced branched-chain amino acid (BCAA) oxidation in muscle, and plasma levels of these AAs might fluctuate with the stage of tumor [22]. Elevated levels of BCAA in plasma have been reported in different types of cancer [23] and have been associated with insulin resistance [20, 24], which is a recognized feature in cancer patients, particularly in late stages with cachexia. In our patients, higher levels of BCAA reflected both the risk of a more severe stage of HNC (isoleucine) and a lower OS (leucine and isoleucine).

It is interesting to note that in our patients we found high levels of aminoadipic acid in the serum of patients with reduced risk of advanced stage of HNC. Aminoadipic acid is a poorly characterized product of lysine degradation, and it may appear in the circulation from degradation of whole tissue or plasma proteins. It has been described experimentally that plasma aminoadipic acid has a role in modulating glucose levels, being augmented as a compensatory response to hyperglycemia, probably by upregulating insulin secretion in early insulin resistance [25].

Serum phenylalanine was highly predictive in our group for both risks of advanced stage and decreased OS. It is generally accepted that phenylalanine plasma concentrations are elevated in cancer, as a reflection of increased muscle proteolysis [26]. Together with BCAA, increased levels of phenylalanine have been observed in head and neck squamous carcinoma cells from different patients [27].

Higher levels of glycine were associated in our patients with decreased OS and with increased risk of advanced stage, while higher serine was significantly associated with decreased OS and, with near-borderline significance, with increased risk of advanced stage. Glycine and serine are closely linked; biosynthesis and uptake of both AAs are usually increased in cancer cells [28], and their relation to one-carbon metabolism is a highly relevant aspect of tumor metabolism for a variety of reasons [29]. Glycine uptake and catabolism promote tumorigenesis and malignancy [30], and its biosynthesis is deemed as a central process in sustaining rapid proliferation [11, 28]; serine serves as a central hub in the metabolic network for many aspects of cancer cell survival and proliferation [28]. Higher concentrations of glycine and serine have been detected in the malignant head and neck squamous cell carcinoma tissue samples compared to surrounding normal tissues [15, 31].

Also, increased levels of 4-hydroxyproline and ethanolamine reflected the risk of a more severe stage of HNC and of a lower OS. 4-Hydroxyproline production from proline is quite critical for tumor survival by stabilizing HIF-1alpha (hypoxia-inducible factor-1 alpha) under hypoxia. Clinically, elevated HIF-1alpha levels in a number of cancers, including HNC, have been associated with aggressive tumor progression and thus implicated as a predictive and prognostic marker [32, 33]. Ethanolamine is not an AA but a primary amine and a primary alcohol [34], and its clinical significance in human blood has not been clarified [35]. Anyhow, ethanolamine has been shown to stimulate the rapid growth of mammalian cells in culture, and therefore, it has been called a growth factor [36]; it also has an important role in cell proliferation as phosphatidylethanolamine participates in the promotion of synthetic DNA [35, 36].

In our patients, higher levels of beta-alanine and beta-aminobutyric acid, both nonproteogenic beta AAs, were associated with increased risk of advanced stage of cancer. Beta-aminobutyric acid, an isomer of aminobutyric acid, is mainly known for its function in plant disease resistance; its role in human physiology is presently unclear, and its significance in the context of human cancer has yet to be clarified. Increased levels of beta-alanine have been described in oral wash samples of patients with head and neck squamous cell carcinoma compared to healthy controls [37]. Sources for beta-alanine include pyrimidine catabolism of cytosine and uracil [38].

Increased levels of arginine were associated with reduced OS. Arginine is among the AAs for which elevated tumor utilization has been established in various other processes, in addition to protein synthesis. In particular, the rapid proliferation rate of tumor cells also requires enhanced production of bioactive products of arginine metabolism, including polyamines and nitric oxide [26]; hence, modulation of the enzymes arginase and nitric oxide synthase can modulate tumor proliferation. Various malignant tumor tissues contain a considerable amount of arginase which converts arginine to ornithine and urea; ornithine is the precursor of polyamines, which are essential components of cell proliferation [39]. Nitric oxide can play variable roles in tumor growth, being potentially toxic for malignant cells, depending on the stage and biology of tumors [39–41]. Arginine metabolism is therefore highly dysregulated in cancer [42], and the prevailing route determines the fate of arginine in tumor promotion or regression; in patients with oral cancer, significantly increased serum arginase activity and nitric oxide levels have been reported [39]. Since arginine is known to be essentially required for the growth of HNC cells, arginine deprivation has been considered as a potential anticancer approach [43, 44].

Associations between high levels of histidine and lysine and reduced OS were found in our patients. Mukherji and colleagues reported that elevated levels of these AAs were more likely found in head and neck tumor tissue compared to normal tissue [45]. Increased serum levels of lysine have been reported in patients with HNC compared to controls [46]. In our study, high lysine also approached borderline significance for increased risk of advanced stage HNC. With regard to the stage of HNC, the result for histidine appeared at variance with that on OS, and this aspect may deserve further investigation.

Serum 3-methylhistidine levels were higher in our patients with reduced OS. High circulating 3-methylhistidine is considered generally as a marker of muscle proteolysis, and increased levels have been proposed as biomarkers of frailty [47]. Significantly higher levels of serum 3-methylhistidine have been described in patients with HNC compared to healthy subjects [46].

A limitation of our study is that there was no control group of healthy individuals to compare the AA levels against. However, this should not alter the reliability of the described results, as these address, within the whole pool of patient measurements, differences in AA values associated with differences in the stage of disease and in overall survival.

## 5. Conclusion

The significance of our study stems from the scarceness of knowledge regarding changes in serum AAs associated with the stage of disease and prognosis in HNC. We showed some findings which may be generically related to the information provided by previous clinical and experimental studies and totally new findings which deserve deeper assessment. Deeper assessment is also needed to specifically characterize serum AA profiles in HNC patients and their relationship with underlying metabolic changes and already available predictive biomarkers. Characterizations should even address specific landmarks for tumor type, disease stage, prognosis, and the implications for nutritional intervention and other treatments.

## Figures and Tables

**Table 1:** Description of the 202 patients included in the analysis.

Parameter	*N*	%
Age at diagnosis (mean, sd)	63.0	11.4
Gender		
*Male*	152	75.2
*Female*	50	24.8
*Missing*	0	—
Tumor stage		
*Early*	72	35.6
*Advanced*	130	64.4
*Missing*	0	—
Tumor subsite		
*Oral cavity*	54	26.7
*Oropharynx*	31	15.3
*Hypopharynx*	10	5.0
*Larynx*	105	52.0
*Other*	2	1.0
*Missing*	0	—
Treatment type		
*Surgery*	62	30.7
*Chemo or radio*	61	30.2
*Surgery and radio or chemo*	78	38.6
*Missing*	1	0.5
Smoking status		
*Never smoker*	32	15.8
*Former smoker*	124	61.4
*Current smoker*	38	18.8
*Missing*	8	4.0
Alcohol drinking status		
*Never drinker*	47	23.3
*Former drinker*	12	5.9
*Current drinker*	136	67.3
*Missing*	7	3.5

**Table 2:** Odds ratios (ORs) and 95% confidence interval (95% CI) for advanced vs. early stage of HNC.

Amino acid	ORs (95% CI)	*p* value
Alanine	0.47 (0.19-1.12)	0.089
Alpha-aminobutyric acid	**0.40 (0.16-0.98)**	**0.045**
Aminoadipic acid	**0.36 (0.17-0.78)**	**0.010**
Arginine	0.59 (0.32-1.11)	0.101
Asparagine	0.67 (0.37-1.23)	0.195
Beta-alanine	**2.55 (1.17-5.60)**	**0.019**
Beta-aminobutyric acid	**1.91 (1.00-3.65)**	**0.048**
Citrulline	0.68 (0.36-1.27)	0.228
Ethanolamine	**2.25 (1.20-4.24)**	**0.012**
Gamma-aminobutyric acid	nc	nc
Glycine	**2.35 (1.17-4.73)**	**0.016**
Histidine	**0.40 (0.19-0.85)**	**0.018**
Isoleucine	**2.39 (1.20-4.76)**	**0.014**
4-Hydroxyproline	**3.16 (1.02-9.78)**	**0.047**
Leucine	1.65 (0.85-3.19)	0.141
Lysine	2.13 (0.95-4.77)	0.066
Methionine	2.15 (0.73-6.26)	0.163
1-Methylhistidine	0.59 (0.32-1.11)	0.102
3-Methylhistidine	0.77 (0.38-1.58)	0.473
Phenylalanine	**2.72 (1.42-5.21)**	**0.003**
Phosphoethanolamine	nc	nc
Proline	**0.24 (0.09-0.62)**	**0.003**
Sarcosine	0.43 (0.15-1.21)	0.109
Serine	1.76 (0.95-3.26)	0.075
Threonine	0.42 (0.17-1.05)	0.064
Tryptophan	**0.41 (0.22-0.76)**	**0.005**
Tyrosine	0.44 (0.13-1.46)	0.181
Valine	1.30 (0.71-2.39)	0.399

^a^Odds ratio adjusted for age, sex, smoking status, and alcohol drinking status. nc: not computable.

**Table 3:** Predictors of OS among 202 HNC cases by multivariate analysis.

Amino acid	HRs (95% CI)^a^	*p* value
Alanine	0.64 (0.35-1.15)	0.135
Alpha-aminobutyric acid	**0.41 (0.24-0.69)**	**0.001**
Aminoadipic acid	1.13 (0.65-1.94)	0.671
Arginine	**2.13 (1.07-4.24)**	**0.030**
Asparagine	2.76 (0.99-7.74)	0.053
Beta-alanine	0.70 (0.35-1.40)	0.309
Beta-aminobutyric acid	1.04 (0.63-1.72)	0.882
Citrulline	0.65 (0.39-1.08)	0.099
Ethanolamine	**2.28 (1.07-4.84)**	**0.032**
Gamma-aminobutyric acid	nc	nc
Glycine	**2.49 (1.17-5.32)**	**0.018**
Histidine	**2.98 (1.44-6.19)**	**0.003**
Isoleucine	**3.08 (1.49-6.40)**	**0.002**
4-Hydroxyproline	**1.66 (1.00-2.74)**	**0.048**
Leucine	**2.29 (1.17-4.48)**	**0.015**
Lysine	**2.34 (1.19-4.60)**	**0.014**
Methionine	1.40 (0.85-2.31)	0.189
1-Methylhistidine	0.59 (0.35-1.00)	0.050
3-Methylhistidine	**1.83 (1.07-3.13)**	**0.027**
Ornithine	1.56 (0.93-2.63)	0.091
Phenylalanine	**2.06 (1.19-3.57)**	**0.010**
Phosphoethanolamine	nc	nc
Proline	1.63 (0.92-2.88)	0.092
Sarcosine	1.54 (0.91-2.62)	0.107
Serine	**2.71 (1.39-5.31)**	**0.004**
Threonine	0.67 (0.41-1.11)	0.121
Tryptophan	0.68 (0.39-1.19)	0.175
Tyrosine	1.52 (0.82-2.82)	0.187
Valine	1.30 (0.74-2.29)	0.360

^a^Hazard ratio adjusted for age, sex, stage, treatment type, smoking status, and alcohol drinking status; nc: not computable.

**Table 4:** Comparison of amino acid levels among HNC tumor subsites.

Amino acid	*p* value^∗^
Alanine	0.452
Alpha-aminobutyric acid	0.116
Aminoadipic acid	0.071
Arginine	0.447
Asparagine	0.699
Beta-alanine	0.663
Beta-aminobutyric acid	0.764
Citrulline	0.474
Ethanolamine	**0.026**
Gamma-aminobutyric acid	nc
Glycine	0.075
Histidine	0.505
Isoleucine	0.483
4-Hydroxyproline	0.585
Leucine	0.346
Lysine	0.380
Methionine	0.302
1-Methylhistidine	0.300
3-Methylhistidine	0.953
Phenylalanine	**0.003**
Phosphoethanolamine	nc
Proline	0.698
Sarcosine	0.712
Serine	0.087
Threonine	0.306
Tryptophan	0.066
Tyrosine	0.740
Valine	0.369

^a^ *p* value obtained from the Kruskal-Wallis test; nc: not computable.

## Data Availability

The data could be obtained by contacting the corresponding author.
