# Erratum to “Prognostic Role of Serum Amino Acids in Head and Neck Cancer”

**DOI:** 10.1155/2021/5806367

**Published:** 2021-03-19

**Authors:** Gabriella Cadoni, Luca Giraldi, Carlo Chiarla, Jacopo Gervasoni, Silvia Persichilli, Aniello Primiano, Stefano Settimi, Jacopo Galli, Gaetano Paludetti, Dario Arzani, Stefania Boccia, Ivo Giovannini, Giovanni Almadori

**Affiliations:** ^1^Dipartimento di Scienze dell'Invecchiamento, Neurologiche, Ortopediche e della Testa-Collo, Fondazione Policlinico Universitario A. Gemelli IRCCS, Largo Gemelli 8, I-00168 Rome, Italy; ^2^Istituto di Clinica Otorinolaringoiatrica, Università Cattolica del Sacro Cuore, Rome, Italy; ^3^Sezione di Igiene, Dipartimento Universitario di Scienze della Vita e Sanità Pubblica, Università Cattolica del Sacro Cuore, Rome, Italy; ^4^CNR-IASI Centro di Studio per la Fisiopatologia dello Shock e Biomatematica, Università Cattolica del Sacro Cuore, Rome, Italy; ^5^Dipartimento Scienze di Laboratorio e Infettivologiche, Fondazione Policlinico Universitario A. Gemelli IRCCS, Rome, Italy; ^6^Dipartimento di Scienze Biotecnologiche di Base, Cliniche Intensivologiche e Perioperatorie, Università Cattolica del Sacro Cuore, Rome, Italy; ^7^Dipartimento della Salute della Donna, Del Bambino e di Sanità Pubblica, Area di Sanità Pubblica, Fondazione Policlinico Universitario A. Gemelli IRCCS, Rome, Italy

In the article titled “Prognostic Role of Serum Amino Acids in Head and Neck Cancer” [1], authors Carlo Chiarla and Ivo Giovannini were affiliated to “Sezione di Igiene, Dipartimento Universitario di Scienze della Vita e Sanità Pubblica, Università Cattolica del Sacro Cuore, Rome, Italy” which is incorrect. The correct affiliation for both aforementioned authors is “CNR-IASI Centro di Studio per la Fisiopatologia dello Shock e Biomatematica, Università Cattolica del Sacro Cuore, Rome, Italy.” This has now been corrected in the author and affiliation details shown above.

The error was introduced during the production process of the article, and Hindawi apologises for causing this error.

## References

[1] Cadoni G., Giraldi L., Chiarla C. (2020). Prognostic Role of Serum Amino Acids in Head and Neck Cancer. *Disease Markers*.

